# Recent Advances in Gas and Chemical Detection by Vernier Effect-Based Photonic Sensors

**DOI:** 10.3390/s140304831

**Published:** 2014-03-10

**Authors:** Mario La Notte, Benedetto Troia, Tommaso Muciaccia, Carlo Edoardo Campanella, Francesco De Leonardis, Vittorio M. N. Passaro

**Affiliations:** Photonics Research Group, Dipartimento di Ingegneria Elettrica e dell'Informazione, Politecnico di Bari, via E. Orabona n. 4, Bari 70125, Italy; E-Mails: lanottemario@alice.it (M.L.N.); benedetto.troia@poliba.it (B.T.); tommaso.muciaccia@hotmail.it (T.M.); edoardo.campanella81@gmail.com (C.E.C.); francesco.deleonardis@poliba.it (F.D.L.)

**Keywords:** Vernier effect, photonic sensors, silicon photonics, homogeneous sensing, ring resonator, MZI sensor

## Abstract

Recently, the Vernier effect has been proved to be very efficient for significantly improving the sensitivity and the limit of detection (LOD) of chemical, biochemical and gas photonic sensors. In this paper a review of compact and efficient photonic sensors based on the Vernier effect is presented. The most relevant results of several theoretical and experimental works are reported, and the theoretical model of the typical Vernier effect-based sensor is discussed as well. In particular, sensitivity up to 460 μm/RIU has been experimentally reported, while ultra-high sensitivity of 2,500 μm/RIU and ultra-low LOD of 8.79 × 10^−8^ RIU have been theoretically demonstrated, employing a Mach-Zehnder Interferometer (MZI) as sensing device instead of an add drop ring resonator.

## 1. Introduction

The huge demand for precise and accurate detection of harmful gases, biochemical analytes and other several kinds of substances has led to great efforts both by industrial and academic researchers in proposing a large variety of innovative sensors. Several physical phenomena have been employed in order to achieve even better performance, both in terms of sensitivity and limit of detection (LOD). For example, conventional methane gas sensors are typically based on catalytic combustion, involving high working temperatures, low thermal stability and reduced measurement range. In [1], cerium-containing nanostructure elements have been introduced to improve the performance of the aforementioned methane sensors, since cerium possesses high oxygen storage and release ability. Among the huge amount of proposed sensors, optical ones have been demonstrated to be a very intriguing solution in order to achieve high performance with very compact size. A lot of physical phenomena can be effectively employed for sensing, such as absorption [2], fluorescence [3], emission [4] and refractometry [5], to name a few. For example, an all-fibre methane optical sensor is proposed in [6], based on two typical near-infrared (NIR) absorption bands of the detected gas. The authors employed hollow core photonic bandgap fibers (HC-PBFs) in order to maximize the light-gas interaction. However, such a sensor requires a quite complex measurement setup and cannot be integrated with standard readout electronics. In this context, recent advances in Silicon-on-Insulator (SOI) technology have led to the possibility of integrating active and passive photonic devices, such as sources, photo-detectors and optical waveguides, onto the same chip, with a significant performance improvement. Furthermore, SOI technology, as well as other Complementary Metal Oxide Semiconductor (CMOS)- compatible platforms, is a topic of great interest due to the possibility of fabricating multiple sensors and related read-out electronics on the same substrate. This offers evident advantages in terms of miniaturization, robustness, reliability, cost effectiveness and low power consumption. Among optical sensors, evanescent field devices can provide extremely high sensitivity for the detection of harmful gas or biochemical analytes, allowing both real time and label-free detection. A lot of possible integrated optical sensor devices can be found in literature, such as planar waveguide structures [7], Mach-Zehnder interferometers (MZIs) [8] and silicon microring resonators [9], to name but a few.

In this work we review the most recent advances concerning Vernier effect-based sensors, starting from the working principle and the mathematical model, highlighting also the most relevant figures of merit commonly used to describe sensor performance. In addition, we describe some optimization techniques recently proposed in order to fulfill predefined specifications in a particular sensor design. Finally, we investigate a sensor platform based on the Vernier effect and constituted by a MZI architecture suitable for a wavelength-based readout scheme. Numerical results resulting from the mathematical modeling of the aforementioned MZI-Enhanced Vernier Effect are also discussed, focusing on relevant design guidelines.

## 2. Vernier Effect Theoretical Model

In the last few years, the Vernier effect has been demonstrated to be a high performance solution for photonic sensing purposes, allowing enhanced sensitivity and lower limits of detection, compared to more conventional sensing architectures [10–12]. To this purpose, the theoretical model of the Vernier effect is presented in depth in this section, reviewing the state-of-the-art of experimentally demonstrated advanced Vernier photonic sensors. In particular, homogeneous and surface sensing principles are briefly introduced since they have been generally employed in integrated photonics sensors as well as those based on the Vernier effect. Consequently, the operation of photonic sensors based on ring resonators is investigated, focusing on the operation of such devices as constituent building blocks of Vernier effect architectures. Finally, fundamental equations governing the Vernier effect in integrated photonic sensors are determined, highlighting the main figures of merit regarding sensing performance. A detailed analysis of the Vernier effect is presented in [13] too.

### 2.1. Homogeneous and Surface Sensing

Biochemical optical sensors are typically based on the variation of waveguide optical properties due to the presence of a specific analyte close to the sensor surface. A common sensing mechanism exploited for this specific application is the variation of the waveguide effective index, as a function of the concentration of the chemical species to be detected. There are two most relevant sensing mechanisms responsible of the effective refractive index variation: homogeneous and surface sensing [14]. It is well known that when light propagates into an optical waveguide, a certain amount of power is confined into the core region, while the remaining part remains confined in the cladding and substrate regions. The effective index of the propagating optical mode is a function of the optical properties of each region defining the optical waveguide. In particular, the effective index change is deeply related to the amount of light interacting with the analyte, *i.e.*, the confinement factor in the medium where the analyte is concentrated.

Homogeneous sensing can be exploited for the detection of a specific gas or analyte due to a homogeneous variation of the cladding refractive index. A typical example of this sensing principle could be the detection of a gas. In particular, when the photonic sensor works at rest, *i.e.*, in the absence of the gas in the cover material, the cladding refractive index corresponds to the air refractive index. When a gas is concentrated in the cover medium, the cladding refractive index changes proportionally to the gas concentration. If the environment is fully saturated, then the cladding refractive index will be exactly that of the specific gas. Another typical case is represented by glucose sensors, where the optical waveguide is covered by aqueous solution, in which the analyte has to be dissolved. Again, the refractive index will change accordingly to the glucose concentration in the water. The waveguide sensitivity can be evaluated as follows [14]:

(1) $$S_{h}={\frac{\partial n_{\text{eff}}}{\partial n_{c}}|}_{n_{c}=n_{c}^{0}}=\frac{2n_{c}^{0}}{Z_{0}P}\iint_{c}{\left|\vec{E}(x,y)\right|}^{2}\text{dxdy}=\frac{2n_{c}^{0}\iint_{\infty }{\left|\vec{E}(x,y)\right|}^{2}\text{dxdy}}{Z_{0}P}\Gamma_{c}^{I}$$

where:

(2) $$P=\iint_{\infty }\left[\left(\bar{E}\times \bar{H}*+\bar{E}*\times \bar{H}\right)\cdot \bar{z}\right]\text{dxdy}$$

In Equation (1), *Z*_0_ is the free space impedance, *n_eff_* is the effective mode index, *n_c_* is the solution refractive index, $n_{c}^{0}$ is the aqueous solution refractive index in absence of the analyte, and are the electric and magnetic field vector, respectively E̅ and H̅ are the electric and magnetic field vector, respectively and $\Gamma_{C}^{I}$ is the optical field intensity confinement factor in the cladding region, defined as:

(3) $$\Gamma_{c}^{I}=\frac{\iint_{C}{\left|\vec{E}(x,y)\right|}^{2}\text{dxdy}}{\iint_{\infty }{\left|\vec{E}(x,y)\right|}^{2}\text{dxdy}}$$

The integration domain indices, *i.e.*, *C* and ∞, stand for cladding cross section and whole computational region, respectively.

The other sensing mechanism is the so called surface sensing. In this case, the molecule detection is obtained through the immobilization of receptor molecules on the functionalized waveguide surface. When these molecules are captured, a very thin ad-layer grows on the waveguide surface. Since the effective refractive index of the propagating mode is strongly linked to the waveguide geometry, the thicker the adsorbed layer (ad-layer) thickness, the higher the effective index change Δ*n_eff_*. According to the variational theorem, the effective mode index will change as [15]:

(4) $$\Delta n_{\text{eff}}=\frac{n_{m}^{2}-{\left(n_{c}^{0}\right)}^{2}}{Z_{0}P}\iint_{\Sigma }{\left|\vec{E}(x,y)\right|}^{2}\text{dxdy}$$

where *n_m_* is the refractive index of the molecular ad-layer and Σ represents the surface covered by the ad-layer. As previously done in case of homogeneous sensing mechanism, it is possible to define the surface waveguide sensitivity [15] as:

(5) $$S_{s}={\frac{\partial n_{\text{eff}}}{\partial \rho }|}_{n_{c}=n_{c}^{0}}$$

where ρ is the thickness of the molecular ad-layer.

### 2.2. Overview of Ring Resonators

**Figure 1.**
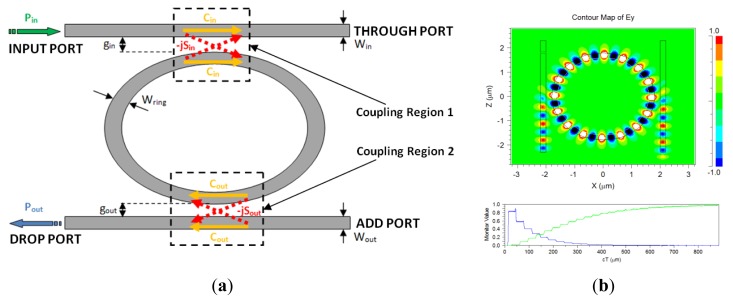
(**a**) Sketch of an Add Drop Ring Resonator; (**b**) FDTD simulation of an ADRR.

Photonic sensors based on ring resonators (RRs) exhibit several interesting advantages compared to other photonic sensors. In particular, the add-drop ring resonator (ADRR) has been selected as the fundamental building block to create more complex architectures based on cascaded multiple ring resonators to exploit the Vernier effect. The conventional planar ADRR working in linear regime is sketched in Figure 1.

In its basic architecture, an ADRR is formed by two optical waveguides, called bus waveguides, serving as input and output ports of the device. These waveguides are designed in order to reach a proper coupling ratio with the ring or racetrack resonator, placed between the two bus waveguides. The resonant condition for the RR takes place when the ring resonator optical length equals integer multiples of the circulating light wavelength, that is:

(6) $$Ln_{\text{eff}}=m\lambda$$

where *L* is the physical resonator length, *n_eff_* is the effective refractive index of the optical mode propagating in the resonator, *λ* is the optical wavelength and *m* is an integer. When the resonance condition is fulfilled, a constructive interference occurs in the resonator, resulting in a buildup of the optical field. The coupling mechanism between bus and resonator is modeled by means of a power coupling coefficient, *k*, defined as the fraction of the incident power transferred from one waveguide to the other one at the end of the coupling length [16]. The coupling mechanism is symmetric with respect to the involved waveguides, so that there is only one coefficient describing the phenomenon.

**Figure 2.**
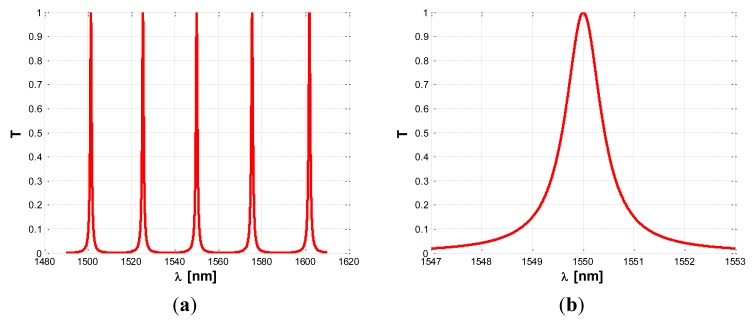
(**a**) Typical amplitude transmittance of an ADRR; (**b**) Detail of transmittance peak.

The amplitude coupling coefficients can be easily calculated from the power coefficient as [16]:

(7) $$\begin{array}{cc} C=\sqrt{\left(1-k\right)}; & -jS=-j\sqrt{k} \end{array}$$

where *j* is the imaginary unit. The amplitude transmittance of an ADRR is defined as the wavelength dependent ratio between the amplitude of the optical field exiting from the drop port and the amplitude of the optical field entering from the input port (see Figure 1). The transmittance can be calculated in the Z-transform domain by means of the Mason's rule for optical circuits, giving as a result [17]:

(8) $$T=\frac{q_{1}q_{2}S_{1}S_{2}\sqrt{\gamma z^{-1}}}{1-q_{1}q_{2}C_{1}C_{2}\gamma z^{-1}}$$

where the q_i_ coefficients take into account the insertion loss of each coupling region, ranging between 0 (infinite insertion loss) and 1 (negligible insertion loss), while the propagation loss inside the ring resonator is modeled by introducing the coefficient γ = e^−αL/2^, where *α* is the loss coefficient per unit length of the optical mode in the cavity and *L* is the resonator physical length. The term z^−1^ in Equation (8) is defined as z^−1^ = e^−jk_0_Ln_eff_^ being k_0_ = 2π/λ, and represents the phase delay in the ring. The subscripts 1 or 2 refer to the coupling region, as indicated in Figure 1. The typical spectral response of an ADRR has a Lorentzian shape periodically repeated along the wavelength axis, as shown in Figure 2.

According to Equation (6), it is evident that only a discrete set of resonant wavelengths, periodically spaced by the frequency free spectral range (FSR), can be coupled out to the drop port of the output bus waveguide. Assuming we work in a wavelength window where the effective index dispersion (due to both material chromatic dispersion and waveguide dispersion) can be well approximated with a first order Taylor expansion, FSR can be calculated from Equation (6) as the inverse of the round trip delay *T_rt_* through the ring:

(9) $$\text{FSR}=\frac{1}{T_{rt}}=\frac{c}{Ln_{g}}$$

where *c* is the light speed in vacuum and *n_g_* is the group index defined as:

(10) $$n_{g}={n_{\text{eff}}(\lambda_{0})-\lambda_{0}\frac{\partial n_{\text{eff}}}{\partial \lambda }|}_{\lambda =\lambda_{0}}$$

In Equation (10) the wavelength λ_0_ is the optical wavelength around which the Taylor expansion is performed. The wavelength FSR is obviously not constant with respect to the wavelength, and can be easily found from Equation (9) to be FSR_λ_=−λ^2^/Ln_g_. FSR_λ_ is a particularly important parameter because it is directly proportional to the dynamic range of the sensor: from Equation (9) it can be inferred that an increase of the dynamic range needs smaller device sizes.

The full-width at half-maximum (*FWHM*) of each Lorentzian shape peak is related to optical losses in the ring cavity and allows to define two important parameters of the device, the quality factor *Q* as in Equation (11) at a specific resonant wavelength *λ*_0_ and the finesse *F* defined as in Equation (12):

(11) $$Q{|}_{\lambda =\lambda_{0}}=\frac{\lambda_{0}}{\text{FWHM}}$$

(12) $$F=\frac{{\text{FSR}}_{\lambda }}{\text{FWHM}}$$

Since the resonant condition depends on the cavity effective index, as in Equation (6), such a filtering mechanism is found to be very suitable in order to perform an optical wavelength interrogation for sensing purposes. In fact, if the ADRR is exposed to the specific substance to be detected, the effective refractive index will change according to Equation (1) or (5), depending on the adopted sensing mechanism. Such a perturbation of the resonance condition will lead to a spectral shift of the drop port transmittance, which usually cannot be measured by means of an Optical Spectrum Analyzer (OSA), due to its limited resolution. To accurately detect this shift, more complex indirect characterization techniques based on the modulation of narrow linewidth coherent radiation sources are required [18]. However, the use of a laser (*i.e.*, light amplification by stimulated emission of radiation) source could be not suitable when measuring large wavelength shifts since an OSA has a much larger dynamic range. The following analytical expression for the wavelength shift can be found from Equation (6) [9]:

(13) $$\Delta \lambda =\frac{\lambda_{0}}{n_{g}}S_{w}\delta$$

where λ_0_ is a reference wavelength (usually coincident with one of the transmittance peak wavelengths) and *S_w_* can be both homogeneous or surface sensitivity. In case of homogeneous sensing, *δ* = Δn_c_ represents the refractive index variation of the cladding medium due to the presence of the detecting substance, while in case of surface sensing *δ* = *ρ* represents the ad-layer thickness. In the remaining part of this paper, the review will focus only on homogeneous sensing, but all the discussed equations can also be referred to surface sensing with straightforward substitutions. A widely adopted figure of merit (FOM) used to evaluate photonic sensors performance is the so called overall sensitivity $S\underset{=}{\text{def}}\Delta \lambda /\text{Δ}n_{c}=\lambda_{0}S_{w}/n_{g}$. It can be seen that such a FOM is given as the product of a waveguide intrinsic characteristic (S_w_)and an architectural dependent feature (λ_0_/n_g_), introduced by the ring resonator.

Although the overall sensitivity gives us significant information concerning the sensor performance, it is still not enough complete to tell us what is the minimum detectable concentration that we are able to measure with that sensor. Such an information is provided by another fundamental FOM, which is the sensor LOD, measured in Refractive Index Unit (RIU) as:

(14) $$\text{LOD}=\frac{{ɛ}_{\text{OSA}}}{S}$$

where *ε_OSA_* is the OSA spectral resolution. To give an example, let us assume a typical gas sensor operating in the near infrared window around λ = 1.55 μm, with a waveguide sensitivity *S_w_* = 0.5 and a typical group index *n_g_* = 4. It is evident from Equation (14) that, to be able to measure a refractive index change of 10^−6^ RIU (typical value for several harmful gas detection applications), the required OSA resolution should be less than 2 pm, which is difficult to achieve, especially in integrated devices.

### 2.3. Overview of Nonlinear Effects in Ring Resonators

There are two main and important reasons to study the nonlinear effects occurring in a SOI microcavity resonator. Firstly, the high values of the enhancement factor occurring in small microcavities induce a reduction of the threshold for the nonlinear effects. In this condition, the nonlinearity could still manifest for relatively low input powers. The second reason is that the presence of the nonlinear effects can induce particular features in the resonant spectra, opening the possibility to improve the performance with respect to the sensor based on resonators operating in linear regime.

Without any lack of generality, it is assumed that the electric field inside the microcavity is predominantly a single transverse mode, *i.e.*, a quasi-TE (dominant horizontal component of electric field) or quasi-TM (dominant vertical component of electric field) polarized mode. Thus, according with the full-vectorial nonlinear coupled mode theory [19], the equations describing the wave propagation in presence of nonlinearity effects can be written as:

(15) $$\begin{array}{l} \frac{\partial a}{\partial t}+v_{g}\frac{\partial a}{\partial z}=j\left[\omega_{0}\left(1-\frac{{\left|a\right|}^{2}}{a_{0}^{2}}\right)-\omega \right]a-\frac{1}{2}\frac{1}{\tau }a-\frac{1}{2}v_{g}\alpha^{\left(\text{FCA}\right)}a-v_{g}\left[\frac{1}{2}\frac{\beta^{\left(\text{TPA}\right)}}{A^{\left(\text{TPA}\right)}}{\left|a\right|}^{2}\right]a\\ +jv_{g}\left[\frac{\gamma }{A^{\left(\text{KERR}\right)}}{\left|a\right|}^{2}\right]a+jv_{g}\frac{2\pi }{\lambda }\Delta na+\xi_{p}S_{p}\\ \frac{dN_{c}}{dt}=-\frac{N_{c}}{\tau_{\text{eff}}}+\frac{1}{2}\frac{\beta^{\left(\text{TPA}\right)}}{\hbar \omega {\left(A^{\left(\text{TPA}\right)}\right)}^{2}}(P^{2}) \end{array}$$

where *a*(*z*,*t*) represents the slowly varying field amplitude (function of time, and propagation direction *z*) for the wave inside the microcavity resonator. Thus, the term $\left[\omega_{0}(1-{\left|a\right|}^{2}/a_{0}^{2})-\omega \right]$ indicates the mismatch from the resonance condition of wave propagating inside the resonator, being *ω* the angular frequency of the input wave and *ω*_0_ the resonant angular frequency of the microcavity in linear regime. Finally, the term $\left(1-{\left|a\right|}^{2}/a_{0}^{2}\right)$ indicates the resonance shift due to Kerr effect, being $a_{0}^{2}=A^{\left(\text{TPA}\right)}n_{0,si}/n_{2}$ with *n*_0_*_,si_* and *n*_2_ the silicon linear and nonlinear refractive index, respectively.

**Figure 3.**
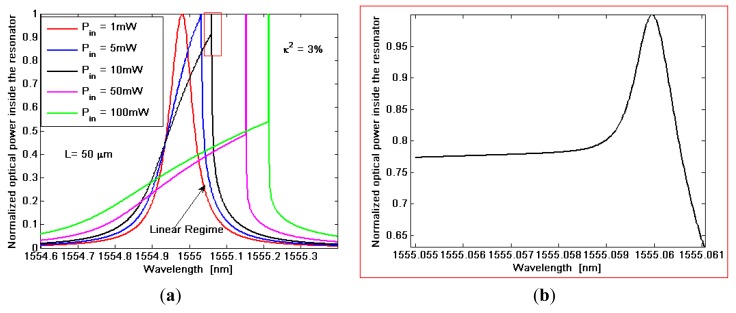
(**a**) Simulated normalized optical power spectra inside the resonator for different input powers; (**b**) zoom plot around the spike for *P_in_* =10 mW.

The term *τ* represents the overall photon decay time of the wave inside the microcavity, related to the resonator quality factor by *Q* = *ωτ* Furthermore, in Equation (15) the terms with *β*^(^*^TPA^*^)^ are due to two photon absorption (TPA), while the coefficients *γ* = *n*_2_*ω/c* take into account the Self-Phase Modulation (SPM) as induced by Kerr nonlinearity. The terms with *α*^(^*^FCA^*^)^ and Δ*n* represent the Free Carrier Absorption (FCA) coefficient and plasma dispersion effect induced by TPA, respectively. The effective modal areas *A*^(^*^TPA^*^)^, *A*^(^*^KERR^*^)^ play a fundamental role since they determine the efficiency by which any nonlinear effect manifests inside the optical SOI waveguide. In the rate Equation (15) governing the free carrier dynamics into the waveguide core, *N_c_* = Δ*N_e_* = Δ*N_h_* is the density of electron-hole pairs generated by TPA process, *τ_eff_* is the effective recombination lifetime for free carriers, *P* is the optical power inside the resonator and *ħ* is the reduced Planck constant. In Figure 3 the normalized optical power spectra inside the resonator for different input powers are shown. Each simulated curve is normalized with respect to its own maximum.

In the simulations the operative wavelength *λ*_0_ = 1,550 nm, a power fraction inside the cavity of 3%, and a cavity length of 50 μm, are assumed. The figure indicates that the nonlinear effects deform the spectrum shape with respect to the typical Lorentzian profile in linear regime. Moreover, the degree of this deformation increases with increasing the input power, starting from the linear regime for *P_in_* ≤ 1 mW. In addition, a very specific feature can be also observed for input powers larger than a determined value, hereinafter indicated as *P̅_in_*. In fact, for *P_i_* ≥ *P̅_i_* the transmittivity spectrum, although significantly deformed, still presents a very narrow, shaped and deep spike, as in Figure 3b. Thus, we can observe that the nonlinear effects induce an increase of the resonant spectrum linewidth related to the increment of optical losses (due to TPA and FCA). On the contrary, the combination of nonlinear and plasma dispersion effects induce the spike formation for. *P_i_* ≥ *P̅_i_* Thus, in nonlinear regime with *P_i_* < *P̅_i_* we have a degradation of sensing performance with respect to the linear regime, whereas, if the resonant sensor works with *P_i_* ≥ *P̅_i_*, the presence of the narrow spike could largely improve its resolution.

### 2.4. The Vernier Effect for Sensing Applications

Nowadays, the Vernier effect is commonly employed in signal processing and telecommunication systems to design optical filters [20] and innovative lasers [21]. The Vernier effect is a well known technique for extending the tuning range of widely tunable lasers containing two reflectors with a different grating period, causing a slightly different peak spacing in the reflection spectrum.

As for optical filters, the Vernier effect is mainly exploited in multichannel WDM systems to select a single wavelength in a broad spectral range (*i.e.*, among a great number of channels), thanks to the large spacing between adjacent resonance peaks in the filter response: a thermally tunable optical filter making use of the Vernier effect has been recently proposed [22]. In case of lasers, the Vernier mechanism has been recently proposed for a compact V-cavity tunable semiconductor laser, based on changing the main lasing mode by shifting the resonant frequency comb of the channel selector cavity [23], and for a single mode III-V/SOI laser, based on reducing the mode competition through the filter RR [24].

**Figure 4.**
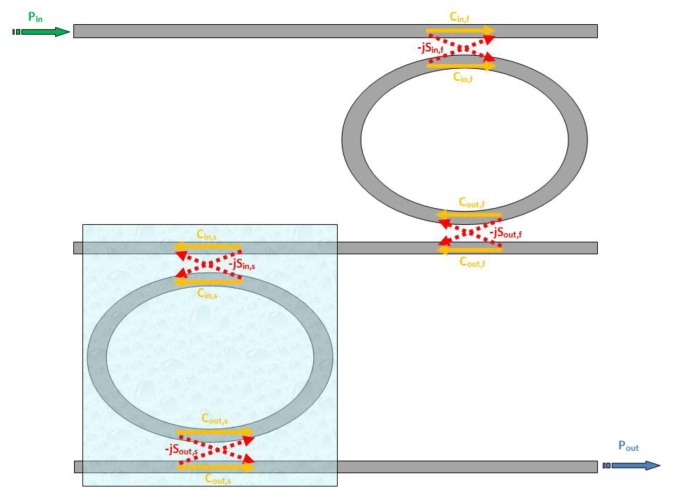
Schematic architecture of a typical Vernier effect-based sensor.

However, in the last few years, several authors have proposed very efficient optical sensors based on the Vernier architecture, demonstrating the possibility to achieve limit of detection even lower than 10^−6^ RIU [25]. In this paragraph the basic expressions for the two previously introduced FOMs are given. The basic architecture of a Vernier effect based sensor is schematically sketched in Figure 4.

The sensor is basically composed by two cascaded ADRRs with different physical lengths, the first one acting as a filter and the second one acting as a sensor. The optical chip is completely covered by a proper cladding medium, except for the windowed sensing region where the cladding is etched in order to expose the sensing ADRR to the substance to be detected. Assuming *T_s_* and *T_f_* to be the transfer functions of the sensor and the filter, respectively, the overall transfer function of the entire architecture will be:

(16) $$T=T_{f}T_{s}$$

As a consequence of their different sizes, the two ADRRs exhibit different FSR and *FWHM*. For sensing purposes, the two ADRRs should be designed to exhibit a common peak at a well defined wavelength *λ*_0_ at rest, *i.e.*, when the detecting substance is not present in the sensing area. In such a condition, the overall transfer function will still exhibit a peak at this precise wavelength.

**Figure 5.**
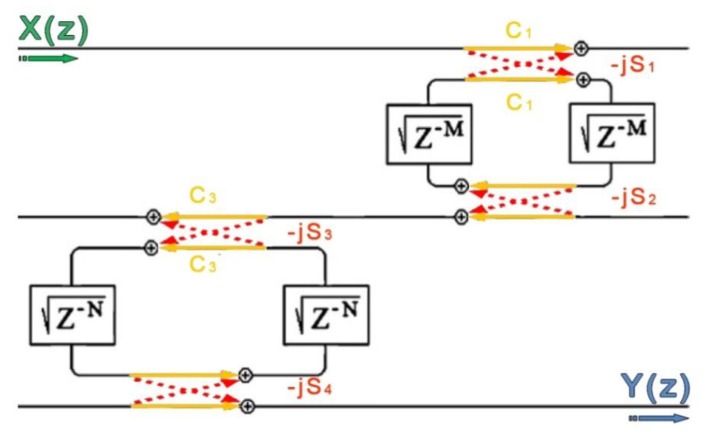
Vernier architecture signal flow graph in the Z-transform domain.

The simple Equation (16) can be rigorously demonstrated by following a generalized approach based on Mason's rule and delay line signal processing, as presented in [25] and sketched in Figure 5. In fact, this method can be applied to every possible configuration employing cascaded RRs (except to those based on concentric rings) which can be assumed linear and time invariant, so it can be applied to the Vernier architecture, too.

The overall transmittance can be calculated according to the following expression, equivalent to Equation (16):

(17) $$T=\frac{T_{f}\Delta_{f}}{1-\left({L_{f}}^{2}+{L_{s}}^{2}\right)+{L_{f,s}}^{2}}$$

where $T_{f}\Delta_{f}=q_{1,f}q_{2,f}q_{1,s}q_{2,s}S_{1,f}S_{2,f}S_{1,s}S_{2,s}\sqrt{\gamma_{f}\gamma_{s}z^{-(M+N)}}$, ${L_{f}}^{2}=q_{1,f}q_{2,f}C_{1,f}C_{2,f}\gamma_{f}z^{-M}$, ${L_{s}}^{2}=q_{1,s}q_{2,s}C_{1,s}C_{2,s}\gamma_{f}z^{-N}$, ${L_{f,s}}^{2}=q_{1,f}q_{2,f}q_{1,s}q_{2,s}C_{1,f}C_{2,f}C_{1,s}C_{2,s}z^{-(M+N)}$, by referring to the same symbolism as adopted for Equation (8). The coefficients *M* and *N* take into account the different time delays accumulated in the two rings. It should be highlighted that, while the filter transfer function is not affected at all by the analyte to be detected, the sensor transmittance experiences a wavelength shift depending on the analyte concentration, according to Equation (13). The change of *T_s_* will obviously reflect to a correspondent change in the overall transfer function. If properly designed, the Vernier architecture will lead to a wavelength shift of the overall transmittance much higher than the shift experienced by *T_s_* alone. This configuration can theoretically operate in two different regimes [25], depending on its geometrical features. The first regime occurs if the free spectral range difference Δ*FSR* = |*FSR* − *FSR_s_*| between the filter free spectral range *FSR_f_* and the sensor free spectral range *FSR_s_* is greater than the full-width at half-maximum (*FWHM*_s_, *FWHM_f_*) of both resonators:

(18) $$\Delta \text{FSR}>\max\left({\text{FWHM}}_{s},{\text{FWHM}}_{f}\right)$$

On the contrary, the second regime occurs if:

(19) $$\Delta \text{FSR}<\min\left({\text{FWHM}}_{s},{\text{FWHM}}_{f}\right)$$

Generally, the first regime is not suitable for sensing applications because the behavior of the spectral response of the whole system cannot be easily related to any change in the analyte concentration. On the other hand, the second regime is very appropriate for high performance sensing. In fact, in this case the Vernier effect can be compared to the well known phenomenon of signal under-sampling. In fact, when the sampling of a sinusoidal signal is performed with a sampling frequency not fulfilling the Nyquist criterion, the signal after the reconstruction filter will be again a sinusoidal wave but with a lower frequency compared to that of the original signal. The filter transmittance acts as a sampling comb, while the sensor transmittance can be thought as the signal to be sampled.

In the second operative regime the overall transmittance exhibits a much larger free spectral range *FSR_tot_* compared to that of both filter and sensor ADRRs, given by [15]:

(20) $${\text{FSR}}_{\text{tot}}=\frac{{\text{FSR}}_{f}\cdot {\text{FSR}}_{s}}{\Delta \text{FSR}}$$

Such an enlargement of the transmittance spectrum involves higher sensitivity and lower LOD. This peculiar feature of Vernier effect based sensors makes them particularly attractive for a number of applications, overcoming the need of high resolution OSAs, quite expensive and not yet suitable for integration on the same sensor chip. Moreover, a wider FSR implies the possibility to achieve a larger dynamic range without drastically decreasing the ring optical length. The overall wavelength shift of the Vernier effect based sensor, Δ*λ_tot_* can be calculated as [16]:

(21) $$\Delta \lambda_{\text{tot}}=\left(\frac{{\text{FSR}}_{\text{tot}}}{{\text{FSR}}_{s}}\right)\Delta \lambda_{s}=\left(\frac{{\text{FSR}}_{f}}{\Delta \text{FSR}}\right)\Delta \lambda_{s}=G_{A}\cdot \Delta \lambda_{s}$$

where Δ*λ*_s_ is the sensor wavelength shift, as defined in Equation (13) and *G_A_* could be defined as the Vernier architectural gain coefficient. It is evident from Equation (21) that if the two ADRRs exhibit a small free spectral range difference, the architectural gain will strongly increase, resulting in a very high overall sensitivity [25]:

(22) $$S_{\lambda ,\text{tot}}=\frac{\Delta \lambda_{\text{tot}}}{\Delta n_{c}}=G_{A}\cdot S_{\lambda ,,s}=G_{A}\cdot \frac{\lambda_{0}{\text{S}}_{\text{w}}}{{\text{n}}_{\text{g}}}$$

In case of Vernier effect-based sensors, particular attention deserves the definition of LOD. In fact, differently from conventional ring resonator based sensors, sensors based on the Vernier effect have an intrinsic digital response [26], meaning that the minimum detectable wavelength shift is exactly the filter free spectral range *FSR_f_*. The limit of detection can be consequently evaluated from Equation (14) by substituting *FSR_f_* instead of the OSA resolution, as:

(23) $$\text{LOD}=\frac{{\text{FSR}}_{f}n_{g,s}}{G_{A}\lambda_{0}S_{w}}=\frac{\Delta \text{FSR}n_{g,s}}{\lambda_{0}S_{w}}$$

where *n_g,s_* is the group index of the sensor ADRR. It should be highlighted that in the near infrared window, for typical resonator lengths (1 μm ÷ 10 mm) the filter free spectral range is always larger than conventional OSA resolutions (∼80 pm), so that using *FSR_f_* in place of ε_OSA_ in Equation (23) makes sense. As expected, the LOD is strongly reduced by increasing the waveguide sensitivity as well as reducing the free spectral range difference.

## 3. Recent Advances in Vernier Effect-Based Photonic Sensors

The first theoretical work concerning the possibility to apply the Vernier effect for sensing purpose has been proposed in literature by Dai in 2009 [11]. In this work, a digital optical sensor is proposed, based on two cascaded ADRRs with slightly different free spectral ranges (FSRs). The aim of the work was to demonstrate the feasibility of chemical sensors employing the Vernier effect. The proposed optical chip is intended to be fully covered by a silica layer, with the only exception of the sensing region, where the silica up-cladding is etched to form a reservoir for the analyte sample. The exposed region is intended to be filled by water, in which the detecting species could be easily dissolved in order to perform an homogeneous sensing mechanism. The ring #1 is chosen as the filter, while the ring #2 (exposed to water) is chosen as the sensor. The proposed sensor is designed in standard Silicon on Insulator (SOI) technology, with 220 nm thick silicon upper layer and 2 μm thick buried silica layer. The optical waveguide employed in the designed sensor is a silicon wire waveguide, 500 nm wide. The chosen light polarization is the TM one, due to the higher waveguide sensitivity [14]. The investigated wavelength window ranges between 1,500 and 1,600 nm. The filtering resonator has a radius of *R_f_* = 38.7475 μm, while the sensing one exhibits a radius of *R_s_* = 39.2728 μm The operative wavelength has been chosen as *λ*_0_=1,500 nm. With such a design, the author finds a filter free spectral range *FSR_f_* = 2.331 nm, and a sensor free spectral range *FSR_s_* = 2.325 nm, resulting in a free spectral range difference Δ*FSR* ∼ 6 pm, which is smaller than the full-width at half-maximum of both filtering and sensing ring resonators. (∼7.4 pm). Because of the different FSRs, the output port will have a spectral response with a major peak and some minor peaks. The major peak of the spectral response from the output port digitally shifts when the effective refractive index of ring #2 changes. As described in Section 2, the shift of the major peak is equal to multiples of the ring #1 FSR. Therefore, the overall device sensitivity results to be G_A_ times higher than that achievable by the device architecture characterized only by the single sensing ring resonator.

**Figure 6.**
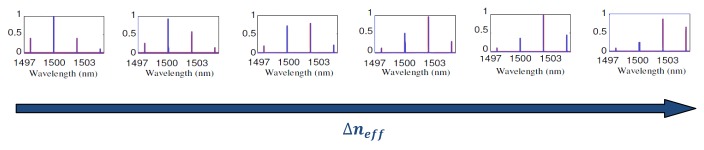
Transmittance spectrum of the proposed Vernier sensor for increasing values of Δ*n_eff_*.

In Figure 6 the overall transmittance spectrum of the proposed sensor is reported, for six different values of effective refractive index changes, *i.e.*, six different concentrations of the substance to be detected. At rest, *i.e.*, when Δ*n_eff_* = 0 the major peak is located at the operative wavelength of 1,500 nm. It can be seen that, if the effective refractive index increases, the major peak tends to reduce, while the adjacent minor peak starts to increase. When Δ*n_eff_* reaches a certain value, the peak located at around 1,503 nm becomes the major one. If Δ*n_eff_* increases further, the major peak will shift to 1,506 nm and so on, resulting in a digital shift of the major peak wavelength. The proposed sensor exhibits a calculated *LOD* = 0.8 × 10^−5^ RIU. Furthermore, by employing resonators with FSR of the order of a few nanometers, the designed digital optical sensor exhibits an ultra-high sensitivity of *S* = 2.91 × 10^5^ nm/RIU, which is approximately two orders of magnitude higher than that of a typical single ADRR based sensor. In conclusion, such a device can be fabricated by using conventional electron-beam lithography (EBL) as well as UV lithography and reactive ion etching (RIE).

Such a result implies the possibility to measure very low concentrations of the detecting substance also using an integrated OSA (eventually with a relatively low resolution), which is a promising solution to realize low-cost, integrated and highly-sensitive, optical sensors on a single chip.

Starting from the theoretical work of Dai [11], Claes *et al.* have experimentally presented in 2010 the first sensor based on Vernier effect [13], characterized by very long resonator lengths. The proposed sensor has been fabricated in standard CMOS compatible SOI technology, with 2 μm thick buried silica layer and 220 nm thick silicon top layer. The physical lengths of filter resonator and sensor resonator have been designed to be 2.528 mm and 2.514 mm, respectively. Then, the two cavities have been designed with folded paths in order to reduce the sensor footprint to 200 × 70 μm^2^. The sensor employs 450 nm wide single-mode photonic wires. The coupling regions have been designed with 6 μm long, straight directional couplers, exhibiting a gap of 180 nm between the two waveguides. The optical chip was fully covered with 500 nm thick silicon oxide layer by means of plasma deposition. Then, a window has been etched in the sensing region by consecutive dry and wet etching, in order to allow the interaction between light and analyte only in the sensing resonator. In this context, the overall sensor has been fabricated in SOI platform with 2 μm buried oxide and 220 nm silicon top layer with CMOS-compatible 193 nm optical lithography and dry etching. Furthermore, a microfluidic channel made in PDMS has been bonded to the optical chip to deliver the liquids to the sensor at a well controlled flow rate. In order to prevent unwanted drifts of the sensor signal, the chip has been temperature-stabilized. Light coupling with the optical chip has been achieved by means of integrated second-order diffractive gratings. The input/output bus is a 10 μm wide ridge waveguide, tapered to a 450 nm wide photonic wire by means of a 150 μm long linear taper. The input light polarization has been tuned by a polarization controller in order to excite only the quasi-TE mode of the waveguides.

**Figure 7.**
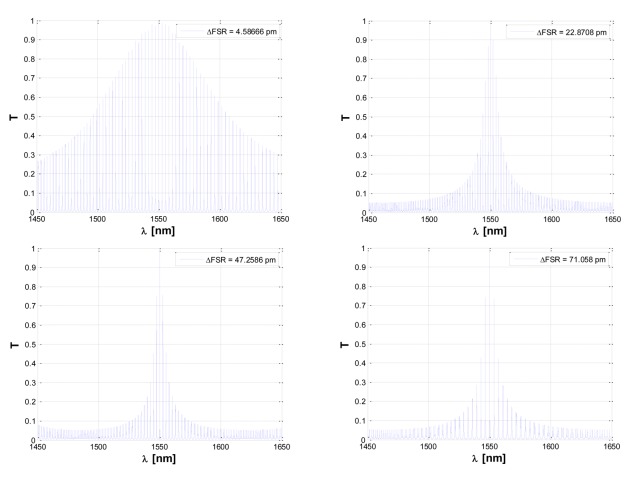
Vernier spectra of the sensor at rest calculated as a function of increasing values of ΔFSR.

According to Equation (23), it can be seen that a very small Δ*FSR* is required in order to reduce LOD as much as possible. Furthermore, as it is clearly demonstrated in Figure 7, the lower the difference between the *FSRs* of cascade-coupled ring resonators, the broader the overall Vernier peak characterizing the transmittance. In particular, it is possible to observe that by increasing the Δ*FSR*, the distance among adjacent spectral lines composing the overall Vernier peak as well as the difference among the central spectral line amplitude and those of adjacent lines, become both larger. Consequently, Δ*FSR* is a crucial design parameter not only for sensing performance but also for the integrated optical readout. In fact, according to Figure 7, small Δ*FSR* values apparently allow ultra-high sensing performance but, in reality, they will generate very broad Vernier spectra, compromising both amplitude and wavelength optical readouts. To this purpose, an interesting readout approach has been proposed in order to overcome this possible limitation [13].

In particular, it has been theoretically demonstrated that, if both filter and sensor resonators have the same full-width at half-maximum (FWHM), the peaks of the overall transfer function can be fitted by an envelope curve *T_env_* with a squared lorentzian shape, given by:

(24) $$T_{\text{env}}={\left|\frac{\sqrt{t_{\max,f}t_{\max,s}}{\left(\frac{\text{FWHM}}{2}\right)}^{2}}{{\left(\frac{\text{FWHM}}{2}\right)}^{2}+{\left(\lambda -\lambda_{c}\right)}^{2}}\right|}^{2}$$

where *t_max,f_* and *t_max,s_* are the transmission coefficients at the resonance for filter and sensor resonators, respectively, λ_c_ is the central wavelength of the envelope peak and *FWHM_LOR_* is defined as:

(25) $${\text{FWHM}}_{\text{LOR}}=\frac{2\times \text{FWHM}}{\Delta \text{FSR}}\min\left({\text{FSR}}_{s},{\text{FSR}}_{f}\right)$$

Such a consideration is again well understandable referring to the example of a signal under-sampling, as previously discussed. By this way, the authors suggest to measure the overall transmittance shift by means of a best fitting technique between the experimentally measured transmittance peaks and the envelope curve of Equation (24). The fitting parameter λ_c_, corresponding to the best fitting condition, is assumed to be the central wavelength of the overall transmittance, allowing to calculate the wavelength shift from the operative wavelength at rest.

In the first step, the authors propose to fit a number of the highest peaks of the measured spectrum with a Lorentzian curve, in order to define all the parameters involved in the envelope curve, apart from λ_c_. Each of these curves analytically describes one of the highest peaks of the overall transmittance. Once these curves have been calculated, Equation (24) is employed in order to fit their analytical maxima, thus obtaining the parameter λ_c_. The authors state that a good measure for the smallest detectable wavelength shift provided by this method is the standard deviation on the fitted central wavelength of the envelope peak, which has been calculated to be 18 pm [13]. It is remarkable that this value is an order of magnitude smaller than the distance between the peaks in the spectrum.

To measure the sensor sensitivity, the sensing region has been firstly exposed to de-ionized water and then to three aqueous solutions with different NaCl concentrations. The refractive index of each of these solutions was calculated as in [27]. A sensitivity of 2,169 nm/RIU has been calculated, in good agreement with the theoretically estimated sensitivity of 2,085 nm/RIU. It is remarkable that the sensitivity of the single ADRR sensor is calculated to be 76 nm/RIU, demonstrating the great advantage in employing the Vernier effect for sensing purpose. The LOD of the proposed sensor is calculated to be 8.3 × 10^−6^ RIU, according to Equation (14).

Another sensor employing Vernier effect has been fabricated and presented by Jin *et al.* [10]. The technology platform is again Silicon-on-Insulator, confirming its importance and suitability in the field of compact and efficient new generation sensors. The authors have employed a standard SOI wafer with 220 nm thick silicon upper cladding and 2 μm thick buried silica layer. The guiding structure was a 1 μm wide ridge waveguide, with a shallow etched ridge height of 20–40 nm, to ensure single mode behavior. The entire device, patterned by contact-photolithography and RIE, was covered by SU8 cladding, apart from the sensing window. The radius of the filter resonator was 120 μm and that of the sensor one 132 μm, giving filter and sensor free spectral ranges of 0.779 nm and 0.709 nm at 1,550 nm, respectively.

According to Equation (14), for TM polarization, the architectural gain is theoretically calculated to be approximately *G_A_* = 11, giving a limit of detection *LOD* = 2.2×10^−4^ RIU while the overall sensitivity is calculated to be S = 3,456 nm/RIU. The fabricated sensor was tested in case of TE polarization with aqueous solution containing three different ethanol concentrations, resulting *LOD* = 5.05×10^−4^ RIU and experimental sensitivity of 1,300 nm/RIU, *i.e.*, over an order of magnitude larger than the sensitivity of a typical single ring sensor [28].

**Figure 8.**
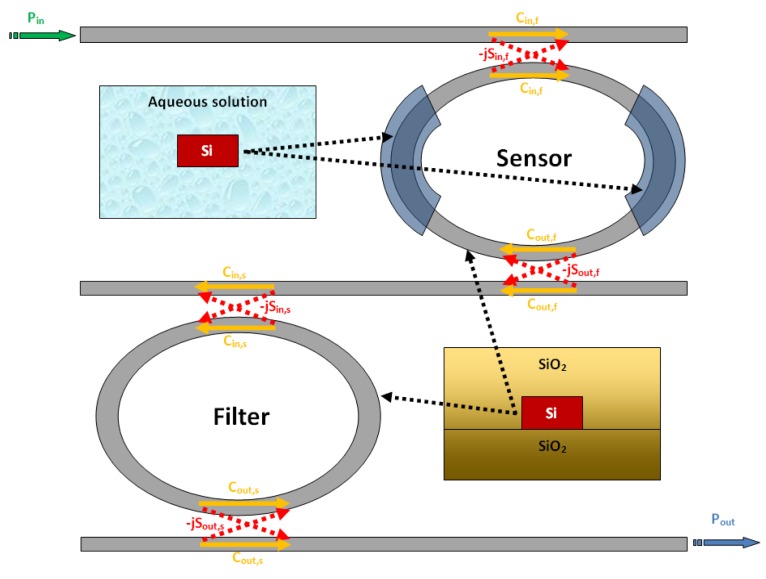
Scheme of the photonic sensor proposed in [12].

More recently, another interesting work concerning a Vernier effect-based sensor has been proposed by Hu and Dai [12]. In this work, the authors proposed to employ suspended SOI nanowires as the guiding structure in the sensing region, in order to achieve very high sensitivity and low LOD. The suspended SOI nanowire was claimed to exhibit a homogeneous sensitivity higher than unity, due to the complete exposure to the analyte to be detected. The sensor is sketched in Figure 8, fabricated in Silicon-on-Insulator technology with a standard SOI wafer characterized by a 220 nm thick silicon upper layer and a 2 μm thick buried silica layer. In particular, the sensor chip was fabricated partially by deep UV lithography. A 600 nm-thick SiO_2_ layer was deposited to cover the whole architecture by using plasma-enhanced chemical vapor deposition (PECVD) technology. Consequently, positive photoresist was used to form the pattern of the sample window. Finally, HF wet etching was used to remove the SiO_2_ upper-cladding as well as the SiO_2_ insulator layer beneath in the widow region resulting in the partially suspended sensing ring. The optical waveguides are single-mode, 500 nm wide silicon photonic wires. In order to maximize the waveguide sensitivity, only the TM mode propagates into the device. The calculated waveguide sensitivity for the suspended wire is as high as 1.19 at an operative wavelength of 1,550 nm. After the definition of the optical waveguides by deep-UV lithography, a 600 nm thick silica layer was deposited by PECVD on the chip surface as a passivation layer. Then, the sensing window was patterned by means of a positive photoresist (two curved regions in Figure 8, with a width of 8 μm).

The suspended photonic wires are then obtained by means of hydrofluoric acid (HF) wet etching. The authors employed a broadband laser as light source of the proposed device, while a low resolution OSA (ε_OSA_ = 100) was adopted as receiver. The input light polarization was tuned by a polarization controller to excite only the TM mode in the optical waveguides, while the light coupling in and from the optical chip was achieved by means of grating couplers. The fabricated chip was tested with four different NaCl concentrations in aqueous solution, while the sensor at rest was exposed only to de-ionized water. The calculated sensitivity of the proposed device was calculated as *S* = 4.6 × 10^5^ nm/RIU, while the limit of detection was *LOD* = 4.8 × 10^−6^ RIU, according to Equation (25).

In a very recent paper [29], another optical device based on cascaded microring resonators has been experimentally demonstrated. The peculiarity of this new proposal consists in the fabrication on a silicon nitride (SiN) platform. Two types of buffer layers, benzocyclobutene polymer and thermal silicon oxide, were tested at two operating wavelengths, 1.3 μm and 1.5 μm, and the experimental results are particularly promising, especially in terms of stability of the Vernier spectrum, for the fabrication of highly sensitive optical sensors in wide operating wavelength range.

**Figure 9.**
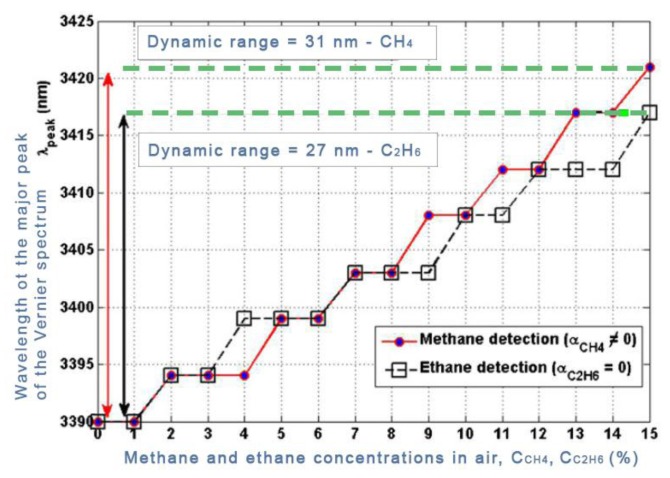
Operative functions of Vernier-based sensors for ethane and methane detection in mid-IR.

In order to fulfill the specifications required by a particular application field, the design of a Vernier based sensor should be optimized to achieve best performances on the base of specific physical constraints related to the context. For example, the design of a photonic gas sensor should take into account the Lower Explosion Limit (LEL) and the Upper Explosion Limit (UEL), *i.e.*, the minimum and maximum concentration of gas, respectively, to set off the explosion in air. A generalized approach for the design of devices exploiting Vernier architecture has been proposed in [25]. The authors present an algorithmic procedure defining a precise sequence of design criteria ending with an optimization loop. The sequential steps suggested by this method include the definition of the technology platform (e.g., SOI) and the waveguide type and architecture (e.g., rib or slot waveguides). Then the operative wavelength *λ_op_* should be chosen, according to the absorption spectrum of the analyte, and propagation losses and the initial set of power coupling ratio between rings and adjacent buses at *λ_op_* should be introduced, too. At that point an approximate set of sizes should be hypothesized on the base of the dimensions of the chip where the device will be integrated (e.g., lab-on-chip). In order to demonstrate the effectiveness and efficiency of the proposed approach, the design of two photonic gas sensors based on the Vernier effect in mid-IR has been demonstrated. In particular, a methane (CH_4_) detector and an ethane (C_2_H_6_) detector have been designed and optimized at *λ_op_* = 3.39 μm and at *λ_op_* = 3.35 μm, as sketched in Figure 9.

Several simulations have been performed to validate the method, considering both homogeneous sensing and optical absorption for methane detection and only homogeneous sensing for ethane detection. A common feature characterizing the response of all photonic sensors based on Vernier effect second regime is observable from Figure 9. Both the devices act as digital optical filters, therefore it is possible to identify a precise number of quantization levels, depending on the specific sensor configuration. Through simulations, sensitivities as high as 224.4 μm/RIU and 218.51 μm /RIU and LODs as low as 1.9568 × 10^−5^ RIU and 2 × 10^−5^ RIU, have been demonstrated for methane and ethane detection, respectively. Several research efforts have been recently done for extending the operation of silicon photonic devices from the near-infrared to the mid-infrared. Nowadays, this intriguing wavelength spectral region is considered a promising operation range for future photonic integrated sensor since a lot of harmful gases (e.g., methane, ethane, ammonia, to name but a few) and chemical/biochemical analytes are spectroscopically accessible within this unexplored range.

In this context, a novel silicon-on-insulator rib-slot photonic sensor based on the Vernier effect and operating at the operative wavelength *λ_op_*, has been theoretically investigated [30]. The sensing architecture is assumed to be fabricated on 6-inch SOI wafers with 400 nm-thick silicon layer on 2 μm-thick buried oxide layer, using conventional e-beam lithography and inductively coupled plasma (ICP) etching. In particular, such a device is constituted by two cascaded ring resonators. Moreover, the filtering ring resonator as well as input and output bus waveguides are assumed to be rib waveguides exhibiting propagation losses lower than 2 dB/cm at the operative mid-infrared wavelength *λ_op_* = 3.8 μm. The sensing ring resonator is based on slot waveguide optimized in mid-infrared since it is possible to achieve an higher sensitivity with respect to conventional silicon rib waveguides. Furthermore, a technology solution consisting in a rib-slot modal converter has been also proposed in order to prevent the optical absorption induced by the buried silica layer at the aforementioned wavelength. In addition, propagation loss of such slot waveguides have been experimentally measured being about 2.6 dB/cm, well acceptable for overall footprints of a few μm^2^. In conclusion, the proposed Vernier rib-slot sensor architecture can exhibit wavelength sensitivity as high as 20.6 μm/RIU and LODs as low as ∼ 4 × 10^−4^ RIU for homogeneous sensing.

**Figure 10.**
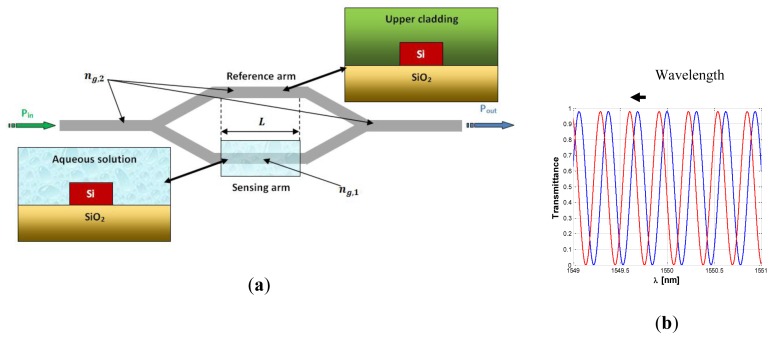
(**a**) Sketch of a MZI sensor; (**b**) Typical transmittance of the MZI sensor at rest (blue curve) and in presence of the analyte (red curve).

As evident from the works which have been discussed so far, the Vernier effect provides a large improvement in photonic sensor performance, concerning both sensitivity and LOD. However, it can be seen from Equation (13) that the ring resonator sensitivity is inherently limited by the effective group index of the optical waveguide. In fact, it should be noticed that the waveguide sensitivity cannot be raised much more than unity, even in case of slot waveguides [14] or suspended silicon nanowires [12]. In the same way, the operative wavelength is typically imposed by optical properties of silicon and available source/receiver apparatus. Typical guiding structures, operating in the near infrared spectrum, usually exhibit effective group indices as high as 4 or even more, which strongly limits the overall sensor sensitivity. In fact, as it is possible to see from Equations (22) and (23), the higher the effective group index the higher the LOD but, at the same time, the lower the overall wavelength sensitivity. To this purpose, a new sensor design has been recently proposed [26], in order to overcome this limitation. The operating principle of the proposed sensor is similar to that previously discussed, with an architectural improvement based on the introduction of Mach-Zehnder Interferometer (MZI) in place of the sensor ring resonator. In this work we provide only the results of the aforementioned analysis. The MZI sensor is sketched in Figure 10.

A wavelength interrogated MZI sensor is obtained by covering all the device with a proper cladding layer, apart from the sensing region placed on one of the two interferometer arms. The sensing arm at rest is often exposed to air, for gas sensing purpose, or to aqueous solution for typical biochemical applications.

When the analyte concentration varies, the typical sinusoidal transmittance of the MZI experiences a wavelength shift (positive or negative, depending on the refractive index difference between the claddings of the two arms). The authors demonstrate that the wavelength shift Δλ*_MZI_* is given by:

(26) $$\Delta \lambda_{\text{MZI}}=\frac{\lambda_{0}}{\Delta n_{g,\text{MZI}}}S_{w}\Delta n_{c}$$

where *λ*_0_ is the operative wavelength, usually chosen as one of the transmittance peak at rest, *S_w_* is the waveguide sensitivity of the exposed waveguide, and Δ*n_c_* is the change of the cladding refractive index of the sensing arm due to the presence of the analyte to be detected. The term Δ*n_g,MZI_* represents the effective group index difference between the two arms of the interferometer [26]. For MZI arms of the same lengths, Δ*n_g,MZI_* = *n_g_*_,1_ − *n_g_*_,2_, where *n_g_*_,1_ is the group index of the sensing arm at rest and *n_g_*_,2_ is the group index of the reference arm.

It is worth noting that Equation (26) has the same structure of Equation (13), with the difference that the denominator of the first equation exhibits the group index difference Δ*n_g,MZI_*, while the second equation exhibits the group index of the ring sensor *n_g_*. In order to compare the performance of ring and MZI based sensors, the authors assume both devices as fabricated with the same optical waveguide, so that the waveguide sensitivity *S_w_* reported in Equations (26) and (13) is the same. Assuming both ring and MZI sensing arm as exposed to the same sample solution, the group index of the exposed waveguides are also the same *n_g_* = *n_g_*_,1_. Furthermore, if both sensors get a transmittance maximum at the same wavelength *λ*_0_ at rest, it is possible to obtain an analytical expression of the overall wavelength shift gain of the MZI architecture compared to a ring resonator:

(27) $$\Delta \lambda_{\text{MZI}}=\frac{1}{1-\frac{n_{g,2}}{n_{g,1}}}\Delta \lambda_{\text{RING}}=G_{s}\Delta \lambda_{\text{RING}}$$

**Figure 11.**
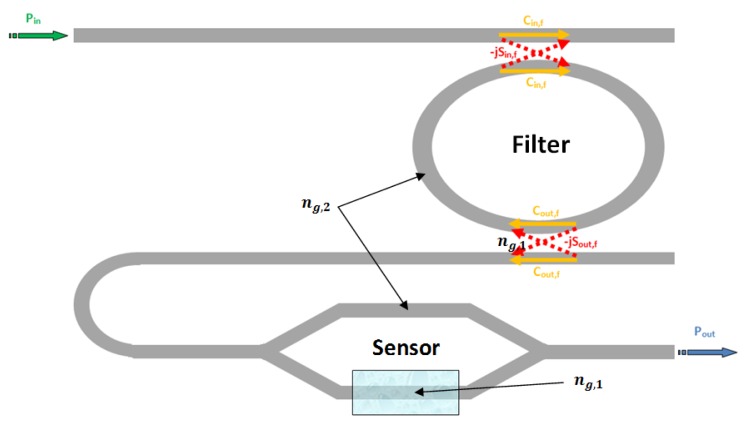
Sketch of a MZI-Enhanced Vernier effect-based sensor.

From Equation (27) it is evident that as the gain coefficient *G_s_* is much higher than unity, than the MZI sensitivity is higher than the ring resonator one. Taking advantage from the relevant result coming from Equation (27), the authors associate the MZI high sensitivity to the architectural improvement based on the Vernier effect, cascading a filter ring resonator (with the same functionality of the standard Vernier effect-based sensor) and a MZI sensor, as sketched in Figure 11.

According to Equation (26), the overall wavelength shift of the Vernier effect is given as the product of the architectural gain *G_A_* and the sensor wavelength shift. In case of MZI sensor, the sensor wavelength shift Δ*λ_s_* is given by Equation (27), so that the overall wavelength shift can be given as:

(28) $$\Delta \lambda_{\text{tot}}=\left(\frac{{\text{FSR}}_{\text{tot}}}{{\text{FSR}}_{\text{MZI}}}\right)\Delta \lambda_{\text{MZI}}=G_{A}\cdot \frac{\lambda_{0}}{\Delta n_{g,\text{MZI}}}S_{w}\Delta n_{c}=G_{A}\cdot G_{S}\cdot \Delta \lambda_{\text{RING}}$$

where the MZI free spectral range (*FSR_MZI_*) is defined as the frequency difference between two adjacent maxima of the MZI spectrum at rest. It is evident that an improvement factor *G_S_* is found for the overall wavelength shift by employing a MZI in place of a ring resonator sensor. Following the same procedure applied for Equation (28), the LOD provided by the sensor [26] is found to be:

(29) $$\text{LOD}=\frac{{\text{FSR}}_{f}\cdot \Delta n_{g,\text{MZI}}}{G_{A}\cdot \lambda_{0}\cdot S_{w}}=\frac{\Delta \text{FSR}\cdot \Delta n_{g,\text{MZI}}}{\lambda_{0}\cdot S_{w}}$$

**Figure 12.**
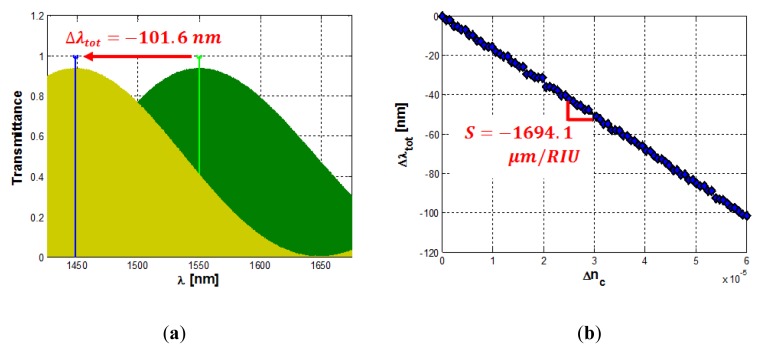
(**a**) Spectral response of a MZI enhanced Vernier effect based sensor at rest (green curve) and in the presence of the analyte to be detected (yellow curve); (**b**) Overall wavelength shift *versus* cladding refractive index change.

A typical spectral response of the proposed sensor is reported in Figure 12a in presence of the analyte to be detected (*i.e.*, yellow curve) and at rest (*i.e.*, green curve). Figure 12a shows an overall wavelength shift Δ*λ_tot_*∼101 *nm* experienced by the transmittance peak for a cladding refractive index change as low as Δ*n_c_* = 6 × 10^−5^ RIU, giving an ultra high sensitivity of S = −1,694.1 μm/RIU, according to Figure 12b. The spectra have been calculated assuming 320 nm wide silicon photonic wires as guiding structure, in SOI technology. The optical chip is assumed to be fully covered by an SU8 upper cladding, apart from the sensing region exposed to aqueous solution. The operative wavelength is fixed to 1,550 nm. In addition, the difference between the *FSR* of the ring resonator (*i.e*., *FSR_Filter_* = 2.791 nm) and that of the MZI (*i.e*., *FSR_MZI_* = 2.771 nm) is Δ*FSR*=19.8 pm, resulting in an architectural gain *G_A_* = 140.97. This set of parameters has been determined by optimizing the whole MZI-Enhanced Vernier architecture. By this way, the limit of detection *LOD* and the wavelength sensitivity S can be maximized as well. However, the optical noise introduced by the optical source, the experimental environment as well as the optical readout scheme (e.g., OSA reflections) will affect experimental measurements and real sensing performance.

The calculated limit of detection for this sensor is as low as *LOD* = 1.84 · 10^−7^. It is worth noting that such not optimized sensor still exhibits a sensitivity approximately three times larger than that presented in [12], with a LOD more than one order of magnitude lower.

In [26] the authors have also given guidelines for an optimal design of the Mach-Zehnder enhanced Vernier effect based sensor both in case of gas sensing and in case of biochemical sensing with aqueous solution cladding. In particular, CO_2_ and ammonia sensors have been designed with several design specifications. In Table 1 we report, for example, the design specifications for the CO_2_ sensor, where *FWHM_tot_* is the full-width at half-maximum of the overall transmittance spectrum, $\Delta n_{c}^{\mathrm{MAX}}$ is the maximum value of Δ*n_c_* to be measured and *L_MAX_* is the maximum acceptable length of the MZI arms. The optimization is carried out, for three different cladding materials, in order to minimize the MZI length as much as possible, always fulfilling each of the design specifications given in Table 1. Finally, it should be highlighted the ultra low LOD and the ultra high wavelength shift achieved for each of the proposed designs.

The review on Vernier-effect based photonic sensors, is now completed by briefly introducing some other few examples of innovative architectures. An ultrahigh-sensitivity silicon double-ring sensors has been presented [31]. Basically, the authors have introduced the idea of cascading two double-ring devices based on the Vernier effect, for realizing simultaneous detection of multiple species. In fact, each of the sensors shares a common bus waveguide for input and reference output. Such sensors can be interrogated by wavelength or intensity readout schemes, and represent a very intriguing solution for low-cost array application towards the fabrication of future photonic Lab-on-chip. In fact, sensitivities as high as 24,300 nm/RIU and 2,430 dB/RIU have been experimentally demonstrated by wavelength and intensity interrogation, respectively.

In [32] a novel Vernier architecture has been proposed by introducing contra-directional grating-coupled racetrack resonator exhibiting the Vernier effect couplers. Theoretical and experimental results show the elimination of FSR (in both drop and through ports) as well as the improvement in the interstitial peak suppression at the drop port and the improvement in the through port insertion loss. These results are due to the suppression of one of the resonances in the through port, which is a consequence of the small bandwidth of the contra-directional grating coupler. Finally, experimental results show an interstitial peak suppression of 29.3 dB.

Moreover, a novel architecture has been proposed in [33], whose name is PANDA ring resonator. It is a modified add-drop filter which consists in a single ring resonator with two lateral nonlinear ring resonators. By this way, the FSR is expanded (in the order of magnitude of terahertz and micrometer) without changing the waveguide ring radius, since it is determined by the least common multiple of the FSRs of the individual ring resonators. The authors have also proposed the application of such an architecture for measuring a force in microscale by detecting the overall wavelength shift.

## 4. Conclusions

The present review explains features, performance and advantages of a particular class of photonic integrated sensors exploiting the Vernier effect, with specific reference to the silicon technology platform. In order to introduce this issue, a summary overview of photonic sensors technology based on ring resonators and its application fields is proposed.

Firstly, a detailed theoretical model of their working principle is given, starting from two general sensing mechanisms, homogeneous and superficial one, on which a wide spectrum of photonic devices are based. Then, the main equations describing the behavior of a ring resonator are clarified and the definition of its most important parameters and figures of merit is given. At that point, the Vernier sensor architecture, involving two ring resonators, is illustrated and a comparison with a single ring resonator device is developed in order to highlight the important advantages introduced by this effect.

Finally, some highly accurate sensors based on Vernier effect, chosen from the most intriguing ones recently proposed in literature, are presented in order to show the complete state-of-the-art of this class of devices.

## Figures and Tables

**Table 1.** Design Specification for CO_2_ detection.

LOD	<5.5·10^−7^ RIU
(Δ*λ_tot_*) *	<150 nm
*L_MAX_*	10 mm
*FWHM_tot_*	<400 nm
$\Delta n_{c}^{\text{MAX}}$	6·10^−5^ RIU

* calculated with $\Delta n_{c}=\Delta n_{c}^{\text{MAX}}$.
